# Exploring the Types of Social Support Exchanged by Survivors of Pediatric Stroke and Their Families in an Online Peer Support Community: Qualitative Thematic Analysis

**DOI:** 10.2196/49440

**Published:** 2024-03-15

**Authors:** William J A Wright, Charlotte Howdle, Neil S Coulson, Anna De Simoni

**Affiliations:** 1 School of Clinical Medicine University of Cambridge Cambridge United Kingdom; 2 Medical School, Nottingham City Hospital University of Nottingham Nottingham United Kingdom; 3 Centre for Primary Care Wolfson Institute of Population Health Queen Mary University of London London United Kingdom

**Keywords:** child, internet-based intervention, online health communities, peer support, qualitative analysis, rehabilitation, self-help group, self-help, social support, stroke, support groups, thematic analysis

## Abstract

**Background:**

Pediatric stroke is relatively rare and underresearched, and there is little awareness of its occurrence in wider society. There is a paucity of literature on the effectiveness of interventions to improve rehabilitation and the services available to survivors. Access to online health communities through the internet may be a means of support for patients with pediatric stroke and their families during recovery; however, little research has been done in this area.

**Objective:**

This study aims to identify the types of social support provided by an online peer support group to survivors of pediatric stroke and their families.

**Methods:**

This was a qualitative thematic analysis of posts from a pediatric stroke population on a UK online stroke community active between 2004 and 2011. The population was split into 2 groups based on whether stroke survivors were aged ≤18 years or aged >18 years at the time of posting. The posts were read by 2 authors who used the adapted Social Support Behavior Code to analyze the types of social support exchanged.

**Results:**

A total of 52 participants who experienced a pediatric stroke were identified, who posted a total of 425 messages to the community. About 41 survivors were aged ≤18 years at the time of posting and were written about by others (31/35 were mothers), while 11 were aged >18 years and were writing about themselves. Survivors and their families joined together in discussion threads. Support was offered and received by all participants, regardless of age. Of all 425 posts, 193 (45.4%) contained at least 1 instance of social support. All 5 types of social support were identified: informational, emotional, network, esteem support, and tangible aid. Informational and emotional support were most commonly exchanged. Emotional support was offered more often than informational support among participants aged ≤18 years at the time of posting; this finding was reversed in the group aged >18 years. Network support and esteem support were less commonly exchanged. Notably, the access subcategory of network support was not exchanged with the community. Tangible aid was the least commonly offered type of support. The exchanged social support provided insight into rehabilitation interventions and the unmet needs of pediatric stroke survivors.

**Conclusions:**

We found evidence of engagement of childhood stroke survivors and their families in an online stroke community, with peer support being exchanged between both long- and short-term survivors of pediatric stroke. Engagement of long-term survivors of pediatric stroke through the online community was key, as they were able to offer informational support from lived experience. Further interventional research is needed to assess health and rehabilitation outcomes from engagement with online support groups. Research is also needed to ensure safe, nurturing online communities.

## Introduction

More than 400 children are diagnosed with stroke in the United Kingdom each year [1]. Despite this sizeable number, diagnosis is often a shock to parents because there is little awareness that strokes can affect children [2]. Recovery from pediatric stroke is a long process, with impairments lasting decades after the event [3]. Novel deficits may present many years after the stroke itself, and existing issues evolve and become more numerous as the survivor grows, with as many as three-quarters of families experiencing at least 1 unmet need after stroke [4]. Pediatric stroke is underresearched [5], and as a result, little is known and available to survivors and their families to support them during recovery [2,4,6,7]. Finally, during the recovery process, survivors and their families feel lonely and isolated from other members of their family, previous friends, peers, and other survivors [3]. Peer support groups may be well placed to alleviate these barriers by providing a space where users can share information, advice, and support each other.

A survey of internet access in the United Kingdom conducted in 2020 revealed that the internet is available in the homes of 93% of the population [8]. This provides an opportunity for individuals to connect online and has led to the formation of online peer-support groups for health care conditions. The advantages of having peer support on an online platform have been outlined by a number of studies [9-12]. These include transcending geographic or temporal boundaries to communication, allowing users to interact regardless of where they live and their time commitments. In addition, posts are written anonymously and asynchronously, allowing participants to speak frankly about their experience without fear of being recognized and, furthermore, spend time composing their posts rather than rushing into a response.

A systematic review found that the roles of online peer support groups for long term conditions consist of a shared social identity, learning from the experiences of others, fostering personal growth, and supporting others [13]. Emerging quantitative data shows that online social support groups may have a therapeutic benefit for some users [14], and a systematic review of social networking site interventions for health care conditions found that they had a positive effect on health-related behaviors [15]. A quantitative study on the use of an online peer support group for women with breast cancer found that the group membership had a moderate effect on reducing depression scores, perceived stress, and cancer-related trauma [16].

The role of in-person peer support groups for adult stroke survivors has been evaluated. A systematic review found they offered a platform for shared experience, social comparison, vicarious learning, and mutual gain [17]. Qualitative reviews additionally found that peer support groups for adult stroke were a place where people shared knowledge [18], felt they belonged [18,19], found a purpose in mentoring each other [18,19], and formed and maintained friendships outside the group setting [18,19]. A study looking at the uses of an online forums for stroke survivors found that users shared a story, requested information and/or support, and provided information and/or support [20]. Adult stroke survivors and their families received information and support on an individual basis from an online forum with 95% of user intentions being met [20].

There is a growing literature evaluating the types of peer support offered in a variety of patient groups with chronic conditions using the Social Support Behavior Code (SSBC) framework and the adapted SSBC [10,21,22]. The SSBC measures 5 types of social support: informational support, emotional support, esteem support (expressing respect and confidence in others), network support (sharing a feeling of belonging to the group), and tangible assistance (providing or offering help). Studies using the SSBC to look at the types of support offered on online forums found the same broad pattern. Emotional and informational support were most commonly exchanged. Esteem and network support were less common, and tangible assistance was rare [9,10,22-24]. Online health communities allow larger degree of personal disclosure, possibly due to anonymity when posting [10].

Exploring the support given by peers in an online stroke community could provide insight about unmet needs with regard to rehabilitation services. Indeed, the “Stroke in Childhood Clinical Guidelines,” published in May 2017, requested research that aimed to identify “rehabilitation interventions in line with the emerging evidence of motor, social, behavioral, and communication sequelae following stroke” [5]. This study aims to analyze whether online peer support was exchanged among survivors of pediatric stroke and what type of peer support was exchanged. A secondary aim is to examine whether time (number of years) since the pediatric stroke was related to engagement and the type of support offered.

## Methods

### Design

A qualitative analysis using the adapted SSBC framework [10] on posts by a pediatric stroke population in an online community.

### Setting

The analysis used posts from the 2004-2011 Talkstroke online forum, a UK-based moderated online forum hosted by the Stroke Association website. The forum was set up as part of the charity website to facilitate communication between stroke survivors and caregivers. In total, the archives included 22,173 posts, written by 2583 unique usernames. A total of 58 participants who had a pediatric stroke were identified from the characterization of individual community users, as reported in a previous study [20]. We further excluded 2 users when analysis revealed their age at stroke was 18 years or older and 4 users because their age at the time of posting was unknown. A sample of 52 users remained, who produced a total of 425 posts that were collected in an Excel (Microsoft Corporation) spreadsheet. Characteristics of stroke survivors, including demographics, employment, education, stroke type, initial impairments as well as impairments at the time of posting, support needs, and independence, were retrieved from within the posts in a previous study [20].

### Data Analysis

WJAW read through all posts to become familiar with the data and patient narratives. Considering the potentially different perspectives of survivors who wrote as adults on their experience of rehabilitation from pediatric stroke and survivors who were written about by their parents, posts were split into 2 categories: whether the pediatric stroke survivor was older than 18 years or aged 18 years or younger at the time of posting. This assessed whether the time since the stroke was related to the amount and type of social support given. Rather than excluding posts that were not relevant (ie, did not include instances of social support), all posts were taken into account for the analysis to give an idea of how prevalent social support exchange was within the discussion threads. WJAW analyzed all posts, and CH and ADS independently coded a random 10% of posts. Posts were analyzed individually, not in the context of other posts in the forum thread. Deductive thematic analysis was applied, as described by Braun and Clarke [25]. The adapted SSBC (Textbox 1) was used as a framework to identify the types of social support provided through engagement with the online forums [10], and results have been reported according to the 5 types of social support: informational, emotional, esteem, network support, and tangible assistance. Coding was discussed with CH and ADS until agreement was reached.

**Social Support Behavior Code, from Coulson and Greenwood [10].**

**Support types and definitions**

- Informational support
  Advice: provides ideas or suggestions for action.
  Referral: refers the recipient to other sources of information or help.
  Situational appraisal: helps reassess or redefine the situation being faced by the recipient.
  Teaching: offers detailed information, facts, or news.
- Emotional support
  Relationship: conveys the importance of closeness.
  Physical affection: offers physical contact, such as hugs and kisses.
  Confidentiality: keeps the recipient’s problem in confidence.
  Sympathy: sorrow or regret for the situation faced by the recipient.
  Understanding or empathy: expressions of understanding of the situation or discloses similar experience in a way that conveys understanding.
  Encouragement: provides the recipient with hope and confidence.
  Prayer: offers prayer for the recipient.
- Esteem support
  Compliment: says positive things about the recipient.
  Validation: provides agreement with the views of the recipient.
  Relief of blame: alleviates any feelings of guilt the recipient has about the situation.
- Network support
  Access: provides the recipient with access to new people.
  Presence: offers to be there.
  Companions: reminds the recipient that there are others who share similar experiences and are available.
- Tangible aid
  Loan: lend money to the recipient.
  Direct task: offers to do a direct task.
  Indirect task: offers to take over a task from the recipient while they are stressed.
  Active participation: offers to join the recipient in an activity.
  Willingness: offers or expressions of willingness to help.

### Patient and Public Involvement

A survivor of pediatric stroke aged 22 years (aged 0 years at the time of stroke and not a member of an online stroke community) was contacted after the initial analysis was completed and read the first draft of the results, providing insightful comments that helped to finalize the analysis and informed the discussion.

### Ethical Considerations

The Stroke Association provided access to the archived forums and gave permission for the data to be used for this research purpose. The data from Talkstroke were stored and accessed through the University of Cambridge Clinical School Secure Data Hosting Service. Users of the forums had previously agreed that their data would become public upon registration within the forums, and there is consensus that internet data that are freely and publicly accessible can be used for research without needing ethics committee approval [20]. In order to protect the identity and intellectual property of forums participants, direct quotes have not been used, despite this being normal practice in qualitative research. Summative descriptions of quotes will instead be used throughout the paper. De Simoni et al [20] report a detailed description of the ethics linked to the research in the Talkstroke archives.

## Results

### Participants’ Characteristics

A total of 41 survivors were aged ≤18 years at the time of participation, contributing a total of 273 posts; 11 survivors were aged >18 years and contributed 152 posts. Most survivors in the group aged ≤18 years took part in the community less than 1 year after their stroke, with the majority of content contributed indirectly through third-party users (31/35 were mothers, 89%). Content from the group aged >18 years was reported firsthand by adult survivors of pediatric stroke (Table 1). The time between stroke and participation in the online community for both groups ranged from 2 weeks to 46 years. Further information about our population can be found in Howdle et al [3].

**Table 1:** Characteristics of the online stroke community participants as identified in the posts.

Characteristics			Frequency
**Participants overall**			
	Total number of participants, n		52
	Age of survivor at time of stroke (years), median (range)		6 (0-17)
	**Posts**		
		Total number of posts, n	425
		Number of posts per participant, median (range)	3 (1-56)
	**Identity of person posting, n**		
		Stroke survivor	17
		Mother	31
		Other (aunt, family friend, cousin, mother, and father)	5
	**Sex of stroke survivor, n**		
		Male	24
		Female	26
		Not stated	2
	**Time since stroke (years), n**		
		0-1	20
		1-2	5
		>2	27
		Not stated	0
**Participants aged ≤18 years at time of posting**			
	Total number of participants, n		41
	Age of survivor at time of stroke (years), median (range)		4 (0-7)
	**Posts**		
		Total number of posts, n	273
		Number of posts per participant, median (range)	3 (1-56)
	**Identity of person posting, n**		
		Stroke survivor	6
		Mother	31
		Other (aunt, family friend, cousin, mother, and father)	4
	**Sex of stroke survivor, n**		
		Male	19
		Female	20
		Not stated	2
	**Time since stroke (years), n**		
		0-1	20
		1-2	5
		>2	16
		Not stated	0
**Participants aged >18 years at time of posting**			
	Total number of participants, n		11
	Age of survivor at time of stroke (years), median (range)		13 (0-17)
	**Posts**		
		Total number of posts, n	152
		Number of posts per participant, median (range)	13 (1-33)
	**Identity of person posting, n**		
		Stroke survivor	11
		Mother	0
		Other (aunt, family friend, cousin, mother, and father)	0
	**Sex of stroke survivor, n**		
		Male	5
		Female	6
		Not stated	0
	**Time since stroke (years), n**		
		0-1	0
		1-2	0
		>2	11
		Not stated	0
	**Participants who stated they were...**		
		Holding a driving license	2
		Not holding a driving license	2
		At university or some university	4
		In full-time employment	1
		In part-time employment	1

### Types of Support Requested by Participants

Posts written by participants at the start of a discussion thread followed a similar structure: an introduction followed by an account of their stroke and recovery up until the time of writing. Participants would then request help or ask for general or specific advice. Within the group aged ≤18 years, nearly half of the posts involved sharing their experience of pediatric stroke.

One parent wrote that they were not sure where to start and explained that their child was born with a congenital heart defect. They described the location of (stroke) damage in the child’s brain. They explained that the progress the child has made has amazed everyone, describing the regaining of functions. The user then requested general help and advice as it was all new (p1, stroke aged 0, 0 years poststroke, mother).

Some posts in the group aged >18 years described their experience with pediatric stroke. In general, rather than sharing their whole story, they shared parts that were relevant to the topic of discussion within a thread. Their aim when participating typically seemed to be to give hope and motivation to other survivors.

A survivor aged older than 18 years wrote, first, to say they felt sorry for the original poster’s loss. They then explained their own story about how their childhood stroke was missed. Since then, they have had many tests in the hope of finding a cause; however, the cause of the stroke was still unknown. They went on to complete their General Certificate of Secondary Education and A-level examinations and were currently studying at university. They finished by writing that strokes do happen in young people and about the need for more societal awareness. They then told the survivor that they too struggled to find anyone to help support their rehabilitation, however, reassuring them they were not alone and to not hesitate to ask any questions (p43, stroke aged 13, 7 years poststroke, survivor).

Reaching out for help occurred in posts by those aged ≤18 years and >18 years. These posts tended to involve asking for advice on dealing with a specific type of symptom.

A survivor aged ≤18 years wrote that her headaches were seriously affecting her everyday life. She followed the doctor’s advice to rest, drink water, and take painkillers, but she found she was resting all the time, and it was affecting her sleep. She apologized for “moaning” and then asked for any ideas (p47, stroke aged 15, 1 year poststroke, survivor).

A user aged >18 years wrote that after 30 years, their eyes were “dizzy.” They then subsequently asked if anyone had medication or other advice to stop this (p15, stroke aged 11, 30 years poststroke).

### SSBC Analysis

About half of the posts contained instances of social support. It was common for posts to contain more than one form of support, for example, a user relating their situation to the recipient’s to show understanding and then providing advice based on their experience of a similar situation.

### Informational Support

All 4 subcategories of informational support were represented (advice, referral, situation appraisal, and teaching). Comparing the 2 age categories, informational support was proportionally offered more by the group aged >18 years than the group aged ≤18 years. Advice was offered on a range of topics. In the group aged ≤18 years, these included therapy types, disability allowance, travel, and not giving up and carving out time as a caregiver. Survivors aged >18 years also gave advice on therapy types and financial aid, but additionally discussed driving and how to be a good caregiver.

One user aged ≤18 years told another participant to look into claiming disability living allowance for children aged 16 years, saying it could save them 17% of the cost of alterations to their home (p6, stroke aged 0, 14 years poststroke, mother).

One user aged >18 years advised a caregiver to take their child out of their comfort zone and make them realize that there is more to life than sitting in a chair. They advised that this would motivate the survivor and be lots of fun (p52, stroke aged 17, 21 years poststroke, survivor).

In the referral subcategory, participants recommended research groups and organizations to each other for support and to aid financial claims.

One user wrote that one good organization for their child’s one-sided weakness was “Hemihelp” (p3, stroke aged 0, 11 years poststroke, mother).

Situation appraisal was common among participants in the group aged ≤18 years. This occurred to help others understand that pediatric strokes occurred more commonly than widely perceived, that the recovery process was different in children compared to adults, and to normalize the grief that parents were feeling for their “lost child.”

A user wrote not to be discouraged about hearing recovery stories of adult survivors of stroke, and that recovery for a child was different, as the plasticity of the brain is a childhood phenomenon (p12, stroke aged 1, 0 years poststroke, mother).

There were several instances of users teaching each other in both groups. Topics involved included how specific medications worked and their dosage, the meaning of medical tests, appointments, and the different types of strokes the disability support available and how to access it, and how to deal with the side effects of stroke.

One user aged >18 years advised another to start a diary to write appointments and other important things in. The user said that this piece of information was invaluable, as it would keep their mind on the stroke and not worrying about forgetting things (p52, stroke aged 17, 21 years poststroke, survivor).

A user explained what a magnetic resonance angiography scan is, saying that it is basically a magnetic resonance imaging scan, but that then they add a dye to show how well the blood is flowing through veins and arteries (p22, stroke aged 5, 4 years poststroke, mother).

### Emotional Support

Emotional support was offered proportionally more by participants aged ≤18 years than >18 years. Support was offered to survivors of stroke in both age categories and to caregivers who wrote on behalf of the survivors. About 5 of the 7 subcategories were seen relationship, physical affection, confidentiality, sympathy, understanding or empathy, and encouragement.

Sympathy was the most displayed subcategory. Messages expressed sorrow for participants’ stroke events, bad news since the stroke, bad experiences, ongoing symptoms, and the struggle to find a cause.

One user wrote that she was sorry to hear about another user’s stroke (p4, stroke aged 0, 0 years poststroke, mother).

Understanding and empathy were displayed in the context of loneliness, fighting the urge to give up, acknowledging similar experiences, symptoms experienced, and difficulty finding travel insurance.

One user wrote they knew what another user meant when saying they felt alone as they had that most of their life. (p36, stroke aged 0, 35 years poststroke, survivor).

A user empathized with another user, writing that they too understood the challenges of dealing with a young child (with stroke) as a single parent (p12, stroke aged 1, 0 years poststroke, mother).

Messages of encouragement were present, often in the form of remarks like “good luck,” “chin up,” or “it gets easier over time.” Most encouragement occurred in the context of helping people start their recovery journey, inspiring people not to give up, or wishing good fortune when attending health care institutions.

A health care worker who previously had a stroke as a child shared her story in the hope it would inspire and encourage people that life does not have to end following a stroke and that with determination you can get on and find other ways of doing things. They finished by stating not to let the stroke win (p39, stroke aged 8, 23 years poststroke, survivor).

The relationship subcategory describes the closeness felt between members of the online community. Users told others they did the best thing by joining the community and expressed joy in finding people who were going through similar experiences.

One user wrote how sorry they were to hear another person’s story but that it was lovely to hear from them as it was difficult to find people in a similar situation. They wanted to be kept up to date about how the other survivors were getting on (p2, stroke aged 0, 2 years poststroke, mother).

Physical affection was displayed through affectionate language and symbols. The most common symbol used was an *x* to send kisses.

One user wrote that they hoped another’s child was well and that their heart truly went to their family, then signed off with their name (p11, stroke aged 1, 1 year poststroke, mother).

Another user wished someone a happy new year, followed by 3 kisses (p49, stroke aged 17, 2 years poststroke, survivor).

### Network Support

Around 2 out of 3 subcategories of network support were identified within the data: presence and companions. The most common incidences of presence, a member offering to be there for another member, were related to 2 users finding themselves in a similar position. This prompted them to stay in touch, especially when somebody was offering advice, to see if it worked. Adult survivors of childhood stroke also offered to keep in touch with younger participants in the long term, to keep answering questions related to their recovery.

A survivor aged >18 years wrote that a user could contact them with any questions and they would do their best to answer them (p49, stroke aged 17, 2 years poststroke, survivor).

A mother of a survivor wrote to another to stay in touch; a few mothers had found each other, and it did help (p12, stroke aged 1, 0 years poststroke, mother).

The community offered a lot in the way of companionship. Users reminded each other that everyone on the site had been through similar experiences and were all there to support each other. Users were often complimentary about others in the community.

One user stated that if another felt down, then they could just talk to those on the site, as everyone was there for each other (p36, stroke aged 0, 35 years poststroke, survivor).

A user prompted another to keep posting because the people on the site were brilliant (p26, stroke aged 9, 0 years poststroke, mother).

### Esteem Support

All subcategories of esteem support were present: compliments, validation, and relief of blame.

Compliments were exchanged to thank people for advice and recovery ideas, for sharing their stories, to motivate caregivers, and to congratulate people for joining the community.

One user wrote to another that they admired their confidence and how they wanted to thank them for always being on the site with advice and support (p26, stroke aged 9, 0 years poststroke, mother).

Another user complimented another by saying they thought the rehabilitation measures they had in place were a brilliant idea (p22, stroke aged 5, 4 years poststroke, mother).

Validation was given when survivors had been through similar experiences. Notable themes within validation were missed or delayed diagnoses, poor awareness of pediatric stroke, isolation, feeling tired, and wanting live chatrooms.

One user agreed with another about the difficulty of convincing doctors to do investigations for stroke. They said their child had all the signs of stroke, but they only obtained a computed tomography scan by pushing the consultant hard, who then agreed to get a radiologist out of bed (p12, stroke aged 1, 0 years poststroke, mother).

Another user agreed that previous friends found it difficult to comprehend how difficult recovery was for stroke survivors (p28, stroke aged 9, 2 years poststroke, parents).

There was a single account of relief of blame, reassuring a survivor that they were not a burden and that everyone needs support at some point (p12, stroke aged 1, 0 years poststroke, mother).

### Tangible Aid

Tangible aid was the least common type of social support offered. Loans, direct tasks, indirect tasks, and active participation were not identified within the data set. However, members displayed a willingness to help wherever possible.

One user wrote that they would be more than happy to help if required (p49, 17 years, 4 years poststroke, survivor).

### Patient and Public Involvement

The analysis was read by a survivor of multiple childhood strokes while aged <1 year, who is now studying at university. She commented that she and her family had never had any experience with peer support, whether in person or online; support had only been provided by medical professionals. However, she thought peer support would have been useful for her and her family. She especially valued the teaching and situational appraisal subcategories in informational support, emotional support, and the validation subcategory in esteem support. Some extracts of her comments are reported in Textbox 2.

**Patient and Public Involvement representative’s specific comments on the study results.**

**Regarding situational appraisal, the survivor said:**

- “I know my family were told this a lot while we were waiting to see if my stroke at <0 years would affect me in my first decade. It was emphasised to be aware of the difference between childhood and adult stroke and not to compare the speed of recovery.”

**Regarding teaching, the survivor said:**

- “I am sure this support would have been invaluable to my family if these forums existed when I was born. Instead informational support was given by doctors only.”

**Regarding emotional, the survivor said:**

- “Again I’m sure this emotional support would have been of great help especially in the uncertain times following a stroke, especially since my family were unaware and unable to connect with any of other families who had experienced pediatric stroke. Additionally, emotional support would have been a great help during the early years when there was uncertainty about the future implications of the stroke as it’s apparently hard to judge the extent of the effects, they wait to see if the child walks, talks or can communicate and learn at school.”

**Validation with regards to medical professionals being less experienced with diagnosing pediatric strokes:**

- “I’m sure my family can sympathise with this, it took one very persistent doctor/nurse who was adamant I was having a stroke to investigate further.”

## Discussion

### Overview

This study provides qualitative evidence that online peer support, facilitated by the online community, played an important role in meeting the rehabilitation needs of patients with pediatric stroke. Most posts analyzed displayed social support. Discussion threads engaged a range of people: those who were recently recovering from a pediatric stroke, their caregivers and families, and adult survivors of pediatric stroke. Users appreciated finding others they could share their lived experience of stroke with and relate to similar incidences, particularly regarding the difficulty in getting a diagnosis and rehabilitation. They additionally discussed both the lack of specific local support and available resources and support services. All 5 categories of social support in the SSBC were evidenced; emotional support was the most common, followed by informational support. The group aged ≤18 years provided more emotional support than informational support; however, the group aged >18 years provided more informational support than emotional support. This difference may arise as members of the group aged >18 years engage with the community in order to provide informational support based on their own lived experience of decades of rehabilitation. There were instances of network support and esteem support, and tangible support was exchanged the least. There were no instances of subcategory access support. It was noticeable that survivors in the group aged >18 years displayed empathy less often than survivors in the group aged ≤18 years.

There are 3 strengths to this study. First, the online community facilitated communication and discussion between participants who recently had a stroke and users who had a stroke many years before. This brought unprecedented insight into the long-term lived experiences of pediatric stroke recovery and survivors’ unmet needs. Second, the methodological design of analyzing posts on a forums meant that messages were viewed and assessed directly by the researcher. This is a nontraditional method that helps to avoid potential problems such as retrospective self-reports, recall bias, and researchers’ bias. Third, the population that uses the forums might include people who do not partake in traditional research studies, allowing the needs of an underrepresented patient population to be studied [11].

Limitations of the study include the analysis of a single UK-based online peer-support community dated 2004-2011, decreasing the generalizability of the study. Additionally, although messages imply that users appreciated support from others, the impact of the support given was hard to measure; performing interviews with participants would have been more successful in measuring the effect of comments on users. Moreover, the study population may not be representative of survivors who do not have access to a computer, do not know how to use the online forums, and therefore may not be able to participate in the online community, resulting in a potential patient population being missed from this study [16]. Further to this, participants who were adult survivors needed to be able to read and type, abilities sometimes affected by stroke and potentially acting as barriers to participation. This study was not set to explore the drawbacks of using online forums as support for health conditions, as posts were analyzed individually and not in the context of the thread to which they belonged. This meant we could not analyze how a post affected other users taking part in that thread. Nevertheless, a previous qualitative study of the same stroke online community found that users would promptly counter inappropriate medical information or health behavior [20].

The ability to access support is a form of social medical capital, defined as the “advantages that any user (patient or caregiver) can gain from participation in the social networks provided by online health communities” [26]. This confirms findings from similar studies across a variety of chronic conditions [9,10,23].

Only half of the posts analyzed in the study contained instances of social support. This was much lower than previous studies, which found rates between 83.8% and 98.9% [9,24]. This could be for a number of reasons: there is a paucity of literature for survivors of pediatric stroke to access, resulting in families and survivors having many questions during their recovery. It is possible that participants were asking more questions than they were able to answer. Also, the forum was for survivors of both adult and pediatric stroke, and it is possible that the adult stroke survivors provided support, which was not included in this study sample. Similar to other studies, informational and emotional support were the 2 most commonly exchanged categories of support; network and esteem support were the next most common, with tangible aid being the least common form of support received [9,10,22-24]. This may be reflective of the actual online platform as the means for providing support, where the geographical limitations of the group mean that the ability to perform tasks to help others is limited.

Informational support involved participants giving each other practical and recovery advice. In addition, participants taught each other strategies for dealing with things referred them to resources and helped them appraise situations. Emotional support was commonly offered; participants exhibited affection for each other, and the forums appeared to provide a safe space to share experiences where users could sympathize, empathize, or encourage each other. Network support was present on the forums; there were many messages that expressed a sense of comradery, reiterating that members of the site were there to support each other. This could be particularly useful in the context of pediatric stroke, where the incidence is relatively low and the chances of meeting another survivor who lives close by are low. Esteem support was provided to thank people for their contribution to the site as well as validate people’s recovery processes.

Notably, there were no examples of the access subcategory. Access is defined as providing “the recipient with access to new people.” This is contrary to other studies [9,10,22,23]. This may be another illustration of the low incidence of pediatric stroke, and so not many survivors and families are well known. In addition, medical and public awareness of pediatric stroke in society is low [2,3,7] and as a result, there are very few people and sources of literature available for survivors. There were no examples of confidentiality, a finding found in other similar studies on online forums; this was as expected as posts were made on a public platform [9,10].

A new finding from this study is that posts made by users whose child was aged ≤18 years at the time of posting offered emotional support more commonly than informational support. However, survivors who were aged >18 years at the time of posting gave more informational support than emotional support. This finding can be interpreted in the context of the theory of optimal matching [27], which hypothesizes that specific types of social support may be beneficial in aiding specific types of stress. According to this theory, the controllability of a situation plays an important role in determining what kind of social support will be most beneficial to the individual. Informational support is a type of action-facilitating support that fosters behaviors designed to mitigate a stressor. Emotional support is a nurturing support that helps individuals cope with the emotional consequences of a stressor [22]. It proposes that individuals with controllable problems should benefit most from informational support because they can use this information, advice, and guidance to help them deal with the cause of their difficulties. However, those with uncontrollable problems should benefit more from emotional support because this will help them cope with unpleasant emotions and the stressful negative effects of being in an uncontrollable situation. In the context of pediatric stroke, survivors who are closer to their stroke event may perceive to have less control over events in their life and so offer emotional support. In contrast, survivors aged >18 years may view their stroke event as more controllable and therefore may have perceived that providing informational support from decades of lived experience was more useful. In addition, socioemotional selectivity theory [28] suggests that the degree to which an illness is relatively more chronic or acute could influence the support seeker’s time perspective, whereby chronic illness facilitates problem-focused coping that favors action-facilitating types of support [22]. Pediatric stroke is an illness with both an acute and chronic phase. As predicted by the social-emotional selectivity theory, emotional support is more prevalent in the group aged ≤18 years coping with acute illness, whereas informational support is more common in the group aged >18 years.

### Conclusions

This study brings qualitative evidence that a nation-wide online peer support group was beneficial in drawing support to survivors of pediatric stroke and their families. The community also represented an opportunity for adult survivors of pediatric stroke to validate their experiences many decades post stroke, share information gained through their rehabilitation journeys, provide insight about unmet needs with regard to rehabilitation services, and provide hope to families with more acute stroke incidences. The study additionally highlights that recovery from stroke is a long process, with adult survivors reaching out for advice. More research is needed, in particular interventional research studies, to evaluate the effectiveness of online peer support groups for survivors of pediatric stroke, as well as research to ensure online communities are safe and nurtured.

## Abbreviations

NIHR: National Institute for Health and Care Research
SSBC: Social Support Behavior Code
